# What Do Undergraduates Learn About Alzheimer Disease? An Analysis of Introduction to Psychology Textbooks

**DOI:** 10.1093/geroni/igab046.2805

**Published:** 2021-12-17

**Authors:** Zoe Hancock, Matthew Wynn, Brian Carpenter

**Affiliations:** 1 Washington University in St. Louis, Saint Louis, Missouri, United States; 2 Washington University in St. Louis, Washington University in St. Louis, Missouri, United States

## Abstract

One of the most popular courses for undergraduate students, Introduction to Psychology, is often students’ first exposure to scientific and clinical facts about Alzheimer disease (AD). In order to learn how our current understanding of AD is presented to undergraduate psychology students, we analyzed passages related to Alzheimer disease that appear in contemporary Introduction to Psychology textbooks. We extracted and analyzed passages describing AD from twenty-four best-selling Introduction to Psychology textbooks for both advanced and intermediate undergraduate audiences, published between 2018 and 2020. We applied a standardized coding scheme to the passages to quantify what aspects of AD were most commonly described. Each textbook contained between 1 and 3 major passages regarding AD, most often appearing in the chapters on Memory or Human Development. Average word count for these passages was 409.1 words (SD = 194.8 words). Passages most often covered biological aspects of AD (87.5% of textbooks), symptoms (87.5%), prevalence (75%), and risk factors (75%). Disease prevention (62.5%) and illness course (62.5%) also appeared in the majority of textbooks, while aspects of treatment and management (25%), assessment and diagnosis (12.5%), and caregiving (25%) were mentioned less often. While the majority of books used contemporary and appropriate terminology to describe AD (e.g., “Alzheimer disease,” “dementia,” “neurocognitive disorder”), some textbooks maintained the use of out-of-date and inappropriate terminology (e.g., “senility” or “senile dementia” in 15%). Introductory psychology textbooks provide an opportunity to teach comprehensive, accurate information about AD and publishers and textbook authors could be guided in this effort.

